# Quantophrenia and the Promises of Genetics: Do Research Practices (Dis)advantage the «Conservation» of Species?

**DOI:** 10.1111/eva.70170

**Published:** 2025-10-28

**Authors:** Stéphanie Mariette, Sophie Gerber

**Affiliations:** ^1^ INRAE Univ Bordeaux, BIOGECO Cestas France; ^2^ INRAE Univ Bordeaux, BIOGECO Pessac France

**Keywords:** conservation genetics, environmental impacts, knowledge hypothesis, molecular markers, promises, quantophrenia

## Abstract

Population genetics is concerned with the variability of genetic diversity in populations subjected to different evolutionary forces. One concrete application of this research is international genetic diversity conservation policies. Our perspective manuscript is a plea for research activities and policies that control their environmental consequences, for example, carbon emissions due to technical choices, and are emancipated from the main economic model. We have indeed witnessed a profound transformation in population genetic studies due to the proliferation of molecular markers and DNA sequencing tools. We analyze the underlying assumptions, and even the beliefs, of the scientific community regarding the quantophrenic use of markers when very significant results on the determinants of genetic diversity are already available. We also discuss the implications of these practices for conservation genetics policy at the international level. The community is indeed defending an approach that aims to describe effective population sizes on a large scale, without considering the environmental costs of these actions. In this paper, we also discuss the “knowledge hypothesis,” that is, that knowledge would lead to effective action. We argue that both the meaning (through the associated promises) and the materiality (the environmental footprint of practices) must be considered in order to rebuild the discipline.

## 1 Introduction

The global biodiversity status is grim. The causes of its destruction are now well identified, the human activities playing a major role. Land use explains the ongoing conversion and degradation of habitats (Newbold et al. 2015). Over‐exploitation of species has been described in both terrestrial and freshwater ecosystems (Young et al. 2016). Massive pollution from agricultural and urban activities causes ecosystem imbalances by releasing excess nutrients or toxic products into the environment (Carpenter et al. 1998). Rapidly spreading exotic species have become a major concern on a global scale because they might alter the ecosystem in which they proliferate (Pysek et al. 2020). Climate change, also due to human activities, has at the same time already caused many shifts in species distribution and abundance. The latter effect is expected to increase in the coming decades (Thomas et al. 2004). Of course, active conservation efforts have led to the demographic recovery of certain species, such as large carnivores in Europe (Chapron et al. 2014). However, there is no sign of systematic recovery for other groups of species, such as the entomofauna (Sanchez‐Bayo and Wyckhuys 2019), despite regular pledges by world leaders to reduce the loss of biodiversity (Butchart et al. 2010).

Saving and conserving biodiversity is indeed the goal of many international and national plans and programs. Since 1993, the Convention on Biological Diversity (CBD) has organized the debate at the international level through the Convention of the Parties. The global framework of the CBD addresses the three levels of biodiversity: ecosystem, species, and genetic diversity. Biodiversity conservation is traditionally based on the definition of geographic zones. Integral protected areas are extremely rare. Conservation is usually linked to the maintenance of human activities. For example, ex situ or in situ conservation strategies are used for plant or animal species that are either domesticated or highly exploited by economic activities (forestry).

Recently, a community of conservation geneticists has advocated extending the conservation of genetic diversity to more species (Laikre et al. 2020). They also recommend monitoring populations with appropriate metrics that reflect their genetic status (Hoban et al. 2020). The central idea is to be able to describe the adaptive potential of a given population based on the monitoring of its genetic variation. Estimating the genetic effective population size (*Ne*) then becomes an important goal of this strategy. Conservation policies are hence based on direct counting of individuals or on the use of population genomics tools and methods to infer *Ne*. The relationship between census size and *Ne* is then supposed to sustain conservation policies. Therefore, a great deal of active research is underway to clarify the links between the census size *Nc* and *Ne*, and to determine the impact of spatial structure and life‐history traits on *Ne* estimates (e.g., Fedorca et al. 2024; Parreira et al. 2025). The community of population geneticists consequently believes that their scientific results will enable efficient management and conservation of populations—the subject of this special issue.

The development of conservation policy is accordingly based on scientific knowledge acquired in the field of population genetics. Over the last two decades, this discipline has undergone a profound technical transformation, relying heavily on sequencing devices. This opinion paper aims to analyze the origins of research practices in population genetics and now population genomics. We would like to highlight an obsession with measures and their precision, which is based not only on rational arguments but also on beliefs, as the “knowledge hypothesis.” These researches and political technical choices are blind to their environmental consequences, leading to the invisibility of these topics, or even their invisibilization. Finally, we propose that our community reflects on the need to change current research practices. Our damaged living world needs coherence from us. In conclusion, we present new perspectives based on incorporating ecological economics principles into population genetics, conservation genetics, and policy.

## 2 Population Geneticists Stand on a Mountain of Studies

Theoretical and experimental studies of population genetics, with conservation biology as a potential application, are numerous. The software Structure (Pritchard et al. 2000), which allows for the identification of homogeneous genetic groups, has, for example, been cited more than 25,000 times (Web of Science database source, accessed on 12 February 2025). Since the first use of isozymes, identified with an extraction, a separation with an electrical migration in a gel and its staining (Hunter and Markert 1957), many other biochemical and molecular techniques have been developed, along with associated statistical tools and theoretical developments. Consequently, studies focusing on one species at a time have increased.

Since the 1980s, statistical summaries of genetic studies have been used to describe the structures of genetic diversity. Typically, genetic diversity and/or genetic structure are correlated with taxonomic, biogeographical, ecological, and life history variables collected for each species studied. For example, Nevo et al. (1984) compiled isozyme data in 1111 plant and animal species and showed that genetic heterozygosity was higher in invertebrates than in vertebrates, with a significant effect of body size and geographic range. In a similar approach, only in plants, Hamrick and Godt (1996) analyzed isozyme data sets from 735 species and showed that life form and breeding system significantly influenced genetic heterozygosity and genetic structure. More recent studies using genetic markers in plants also found that the mating system is a key driver of genetic diversity and structure (Duminil et al. 2007; He et al. 2024). These studies also outlined the role of seed dispersal and geographic range or isolation. Using an expanded collection of plant and animal genetic datasets, De Kort et al. (2021) recently found a significantly higher level of expected heterozygosity in animals than in plants, and a significant effect of some life history traits on heterozygosity (animal longevity and size, animal fecundity, and plant dispersal). In the last two decades, analyses and meta‐analyses based on sequence data have also emerged, pointing at the link between life‐history traits and genomic polymorphism (Glémin et al. 2006; Romiguier et al. 2014; Jeon et al. 2024), and more broadly allowing us to investigate the effect, on genomic polymorphism, of variation in effective population size (Ellegren and Galtier 2016).

The genomic knowledge available is thus already abundant. To summarize, meta‐analyses show that life‐history traits are an important explanation for the variation in genetic diversity.

## 3 Acquiring a Large Genomic Dataset Is the New Norm for the Genetic Study of a Species

**FIGURE 1:**
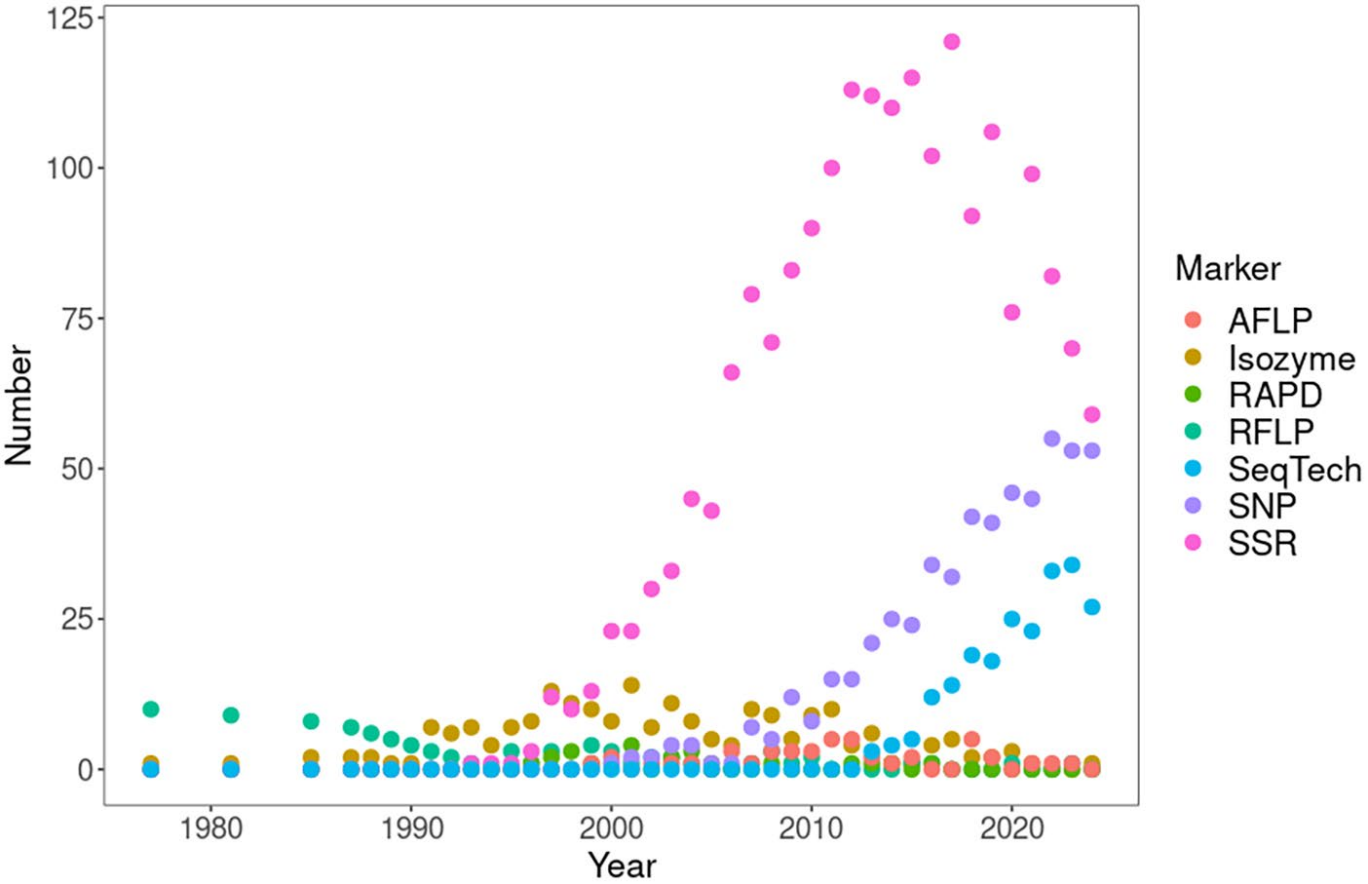
The WoS database was searched by combining a list of marker names together with “genetic diversity.” The figure shows the yearly distribution of the number of citations.

**FIGURE 2:**
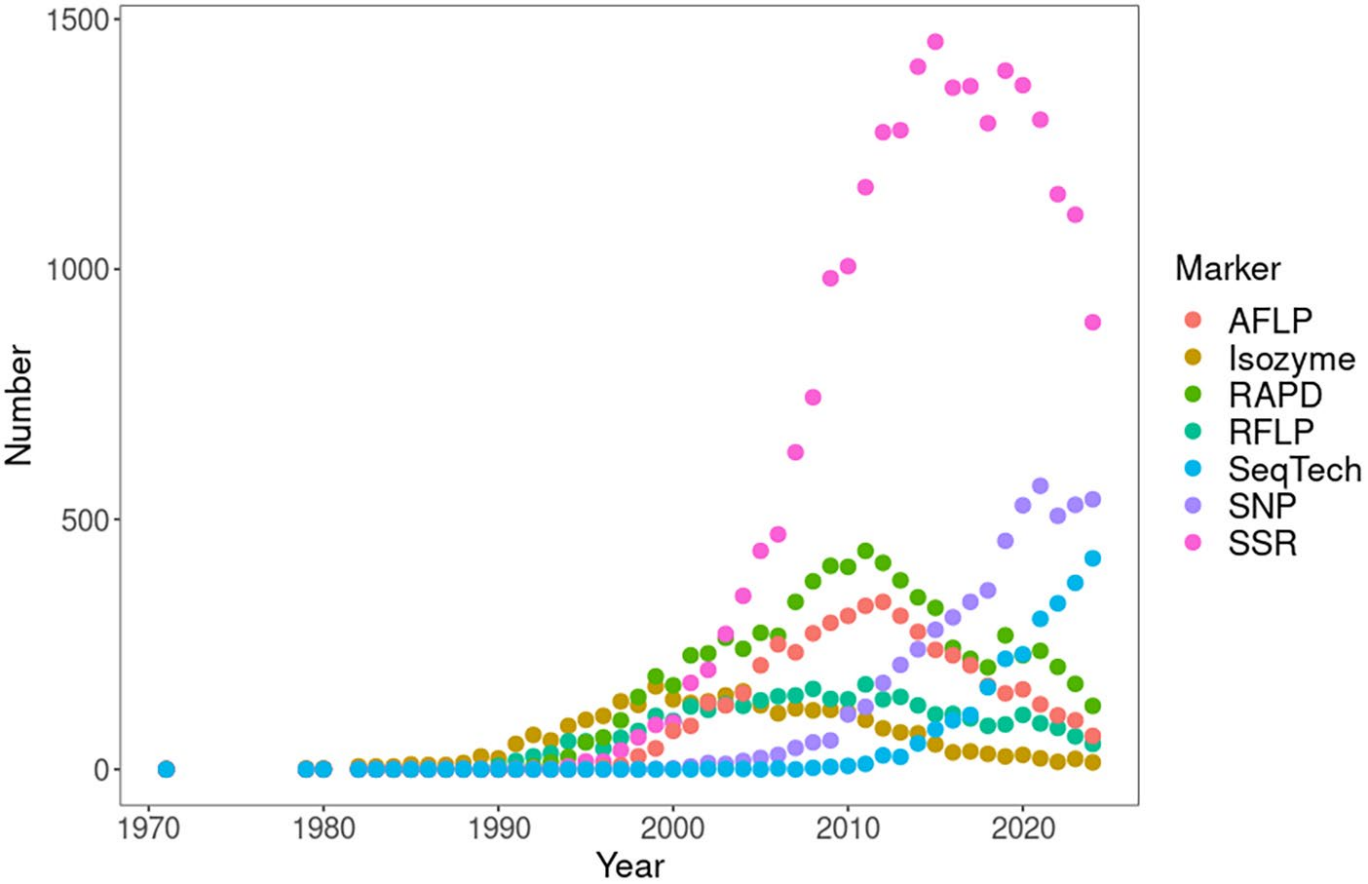
The WoS database was searched by combining a list of marker names together with “effective population size.” The figure shows the yearly distribution of the number of citations.

Literature databases show that single nucleotide polymorphism genotyping (SNP) and sequencing methods (restriction‐site associated DNA sequencing—RAD Seq, whole genome sequencing) have replaced microsatellites in the study of genetic diversity over the last decade (Figure 1). For about two decades, from the end of the 1990s to the end of the 2010s, microsatellites were the most commonly used marker type compared to isozymes, RFLPs, RAPDs, and AFLPs. A similar trend can be seen when each type of marker is combined with the term “effective population size” (Figure 2).

Thanks to this significant technological advancement in population genetics, species can now be analyzed using thousands of markers rather than a handful. Of course, the shift toward these technologies is uneven among species. In particular, knowledge of and access to the whole genome is biased toward certain taxa. For instance, arthropods are underrepresented in animal databases compared to vertebrates (Hotaling et al. 2021). However, high‐throughput techniques such as RAD‐seq allow the discovery of SNPs and genotyping for species for which the entire genome is unavailable (Andrews et al. 2016).

This observed technology replacement is driven by several fundamental scientific justifications. Firstly, large SNP or genomic datasets overcome the limitations of microsatellites or other markers, such as insufficient genome coverage and low data reproducibility (Morin et al. 2004). Secondly, these data can be used for various purposes, not only reconstructing the evolutionary history of species but also studying adaptation and investigating the genetic determinants of phenotypes (Stapley et al. 2010). Lastly, and perhaps more importantly, the thousands, or even millions, of polymorphisms sampled allow many statistics to be estimated with more precision, such as the site‐frequency spectrum. This results in an unprecedented ability to infer the demographic history of species (Lynch et al. 2020). Large access to genomic data has thus made it possible to overcome the lack of precision inherent in the use of a lower number of markers which is partly responsible for inconsistencies when comparing measurements between different marker types (Gerber et al. 2000; Mariette et al. 2002; Nybom 2004). However, increasing the number of markers is not enough when it comes to *Ne*. Using several datasets, Gargiulo et al. (2024) confirmed that large sample sizes are required to obtain an accurate estimate of *Ne*. Moreover, this intensive sampling may not even be sufficient for large populations, for example in forest tree species (Santos‐del‐Blanco et al. 2022).

## 4 International Conservation Genetics Policy Is Based on Research Practices Without Planetary Boundaries

As explained in the preceding paragraphs, international genetics conservation policy is based on scientific expertise and indicators that require intensive marker and/or individual sampling, at the population level, for a given species. This policy does not consider the environmental consequences of technical choices, which need to be addressed.

One of these consequences is the climatic one. The latest IPCC report suggests that “sufficiency”—defined as “a set of measures and daily practices that avoid demand for energy, materials, land and water while delivering human well‐being for all within planetary boundaries”—could help to reduce greenhouse gas emissions (IPCC 2022). There is a growing concern about the carbon impact of science, leading to an increasing number of studies to measure it. The estimate of carbon emissions by the French National Centre for Scientific Research reaches 14.7 t CO_2_ equivalent per person (CNRS 2024). An estimate of annual footprints reaches 10–20 tCO_2_ e/pers. for laboratories in Earth, environmental and space sciences, with the highest footprints for infrastructures, purchases and air travels (Marc et al. 2024). In a more complete survey of 108 different French laboratories, De Paepe et al. (2023) showed that purchases and electricity account for a large proportion of carbon emissions, respectively, 2.3 tCO_2_ e/pers. and 3.5 tCO_2_ e/pers., when modeled for high carbon electricity mixes. This should be considered an underestimate of the climate impact of science due to the low carbon intensity of French electricity (nuclear production). Carbon emissions are particularly high in the fields of science and technology, and life and health sciences. However, even though the community is responsible for large greenhouse gas emissions, acting to reduce them is not the main observed behavior (Blanchard et al. 2022). Discourses of climate inaction exist in the scientific community. The general discourses described by Lamb et al. (2020) are shared by the community, but some specific arguments are developed (Carbou and Sébastien 2023). In particular, scientists may be prompted to justify delays in action by the need to accurately measure the environmental impact of their research. In other words, it seems that scientists have to convince themselves with numbers before they take action. As a result, limited measures are developed in laboratories to seriously reduce the climate impact of a given community.

In addition, the above studies focus on greenhouse gas emissions only, ignoring the impact on biodiversity, water use, or any other component of planetary boundaries as defined by Rockström et al. (2009). The environmental consequences of scientific activities are then underestimated in publications that focus only on the carbon footprint of research.

Like other life science disciplines, the research practices developed in population genomics have an environmental footprint through sampling, laboratory work (DNA extraction, data acquisition, and data production), data analysis using algorithms, models, and statistics, data storage on servers, paper writing, and communication of results involving travels. Although the demand for biodiversity conservation or climate adaptation applications motivates population genomics studies, the impact of the choice of scientific tools is paradoxically overlooked in the discipline. The increased economic accessibility of genomic technologies through falling prices is generally seen as progress, promising to “democratize” these tools for all laboratories. Quoting Hoban et al. (2020): “We note that genetic data are increasingly available and affordable, and government agencies and others are often able to monitor genetic diversity directly, including using historic samples and models of historic genetic diversity. Genomic technologies and analytical methods are rapidly changing, with genome sequencers now the size and cost of a smartphone, and analytical tools able to estimate gene flow and population sizes many generations in the past.” The risk of increasing the community's ecological footprint is missing from these considerations.

In sum, the population genetics community uses the same arguments as scientists who delay action to reduce the environmental impact of their research. Paradoxically, while the community is morally committed with strong promises to save biodiversity (Gerber and Mariette 2023), it is not developing a national or international strategy to drastically reduce its overall environmental impact.

## 5 The Knowledge Hypothesis and the International Research Model Explain Our Quantophrenia

The shift to more data, which we describe in this manuscript, is a very general trend in ecology (Anderson et al. 2021). As previously explained, the accumulation of data is justified by access to more precise measurements. It is also more generally driven by the knowledge hypothesis. *Knowing* more would lead to environmental action. In other words, tackling climate change or better protecting biodiversity would absolutely require better scientific understanding (Dupont et al. 2025). As Devictor (2021, 14) explains, a gradual vision of ecological transition that calls for patience insists on the “weakness of our knowledge, still too immature.” The continuous promise consists in claiming for: “more data, to know more, to develop better policies”—even though the causes of biodiversity loss have been known for decades. Nevertheless, as we have argued before, the knowledge‐action gap lies also within the scientific community itself. Although perfectly aware of environmental issues, it is not acting to massively reduce the carbon emissions and the environmental consequences of its scientific activities.

Related to this knowledge hypothesis, there is a widespread belief that science based on data accumulation is a new way of doing science (Leonelli 2014), and even that new theories would emerge from data‐intensive research. Conversely, it may be desirable to conduct studies that are not solely data‐driven, but that also consider the natural history and fundamental principles of all disciplines related to biodiversity (Anderson et al. 2021). It may be useful to formulate a clear hypothesis before collecting data.

Socio‐economic factors may also explain why scientists continue to accumulate data. The international research system is built on a citation hierarchy, which is part of the social organization of research foundations. Research also follows the dictates of the dominant economic productivist model, as illustrated by the exponential growth in the number of articles between 2016 and 2022 (Hanson et al. 2024). Moreover, Berné et al. (2022) found that air travel—and then the high carbon impact associated with moving—correlates with scientific visibility (publication rate and h‐index). The research model itself leads to climate degradation and the destruction of biodiversity.

As a consequence, “quantophrenia,” the term invented by Sorokin (1956, 103–104), the disease of measurements and numbers, that confounds rigorous analysis with quantification, and wishes to adapt reality to mathematical laws (de Gaulejac 1990), is an adequate way to describe this accumulation of data and publications, and its lack of effect on policy change.

## 6 From Accumulating Genetic Data to Acting: Emancipating Ourselves From Prevailing Assumptions

In summary, there is a widespread belief in the population genetics community that more data describing the genetic status of species and their evolution is needed in order to formulate policies for the conservation of genetic diversity. The correlative increase in the number of markers used in each study is linked to the reaffirmed need to accurately describe populations, either in terms of genetic diversity or effective population size. Recent recommendations from the Convention on Biological Diversity reinforce the need for more data and are actually promoting, in this way, research without planetary boundaries.

We already know the causes of biodiversity loss. As Devictor (2021, 29) outlines: “An urgent need to quantify the state of living organisms and the costs and benefits of protecting them replaces the criticism of a destructive economic model.” Consequently, we hypothesize that describing biodiversity and its decline with more data will not solve the systemic problem facing humanity. We hypothesize that describing more species under the CBD will not improve the global genetic diversity state. More than that, we hypothesize that our quantophrenia is a symptom of a false hypothesis that is distracting us from the socio‐economic underpinnings of biodiversity destruction.

Several lines of reflection can be defined to emancipate the community from dominant assumptions and stereotypes.

One main perspective is to emancipate the discipline and the international conservation policy from the dominant economic model and open it to the principles of ecological economics: integrating planetary boundaries (Fanning and Raworth 2025), rejecting the monetary valuation of biodiversity (Bonneuil 2015), and building a macroeconomic model without growth. One potential negative outcome of such a transition could be that many corporations involved in conservation would either disappear or decide to stop their efforts. However, the inefficacy of the current model in stopping biodiversity loss must be recognized. The monetary valuation of biodiversity leads to intensive efforts focused on specific, emblematic species (Vivien 2022). Monetary valuation also leads to biodiversity compensation politics, whereas no long‐term action is undertaken to actually stop the disappearance of habitats.

A second perspective would be to translate these international principles into national laws, and this would transform environmental policy by directly stopping projects that massively destroy biodiversity.

Finally, all the disciplines necessary for the defense of biodiversity must be implemented and interlinked. If the quantified knowledge of the destruction is not enough to act, it means that other determinants must be taken into account: socio‐economic, of course, but also philosophical or anthropological.

## 7 Conclusion

To conclude, we observe that population genetics and genetic resource conservation models are influenced by the dominant economy, particularly with regard to economic growth. We therefore recommend considering the consequences of this when designing our protocols and formulating conservation policies. In addition, our community of population geneticists, genomicists, and conservationists must recognize its environmental impact, even if this cannot be fully or precisely measured. Studies already suggest that this impact is high, similar to, or even higher than that of the average citizen in an Occidental country. Far from being a call to stop research, this paper is consequently a plea for conservation genetics research that takes the time to build meaningful and robust projects, with rigorous control of its potential environmental impacts. Such studies would test a well‐defined hypothesis, combine fine description of phenotypic traits with precise naturalistic observation of species, and use the necessary polymorphic markers and statistical approaches with robust tools. Acoca‐Pidolle et al. (2024) seem to us one example of such a sober and sound study, linking the effect of declining pollinators to the rapid evolution of the mating system in a plant.

## Conflicts of Interest

The authors declare no conflicts of interest.

## Data Availability

We agree to archive the data associated with this manuscript should the manuscript be accepted.
